# The Role of the Interplay between Stimulus Type and Timing in Explaining BCI-Illiteracy for Visual P300-Based Brain-Computer Interfaces

**DOI:** 10.3389/fnins.2017.00363

**Published:** 2017-06-30

**Authors:** Roberta Carabalona

**Affiliations:** Biomedical Technological Department, Fondazione Don Carlo Gnocchi Onlus (IRCCS)Milan, Italy

**Keywords:** brain-computer interface, semantic categorization, user experience, N1, P300, usability, visual cognition, BCI-illiteracy

## Abstract

Visual P300-based Brain-Computer Interface (BCI) spellers enable communication or interaction with the environment by flashing elements in a matrix and exploiting consequent changes in end-user's brain activity. Despite research efforts, performance variability and BCI-illiteracy still are critical issues for real world applications. Moreover, there is a quite unaddressed kind of BCI-illiteracy, which becomes apparent when the same end-user operates BCI-spellers intended for different applications: our aim is to understand why some well performers can become BCI-illiterate depending on speller type. We manipulated stimulus type (factor STIM: either characters or icons), color (factor COLOR: white, green) and timing (factor SPEED: fast, slow). Each BCI session consisted of training (without feedback) and performance phase (with feedback), both in copy-spelling. For fast flashing spellers, we observed a performance worsening for white icon-speller. Our findings are consistent with existing results reported on end-users using identical white×fast spellers, indicating independence of worsening trend from users' group. The use of slow stimulation timing shed a new light on the perceptual and cognitive phenomena related to the use of a BCI-speller during both the training and the performance phase. We found a significant STIM main effect for the N1 component on P_*z*_ and PO_7_ during the training phase and on PO_8_ during the performance phase, whereas in both phases neither the STIM×COLOR interaction nor the COLOR main effect was statistically significant. After collapsing data for factor COLOR, it emerged a statistically significant modulation of N1 amplitude depending to the phase of BCI session: N1 was more negative for icons than for characters both on P_*z*_ and PO_7_ (training), whereas the opposite modulation was observed for PO_8_ (performance). Results indicate that both feedback and expertise with respect to the stimulus type can modulate the N1 component and that icons require more perceptual analysis. Therefore, fast flashing is likely to be more detrimental for end-users' performance in case of icon-spellers. In conclusion, the interplay between stimulus type and timing seems relevant for a satisfactory and efficient end-user's BCI-experience.

## 1. Introduction

Brain-Computer Interface (hereafter BCI) systems enable people to communicate with others or interact with the environment in a non-muscular way and can serve both as assistive technologies and rehabilitation tools (Wolpaw et al., 2002; Daly and Wolpaw, 2008; Daly et al., 2013) as well as new control interfaces for healthy users (Krepki et al., 2007; Bos et al., 2010). They provide the brain with new output pathways by exploiting changes in end-user's brain activity that result from the execution of cognitive tasks. Data on brain activity are usually non-invasively recorded using EEG, while cognitive tasks can range from mental tasks, such as motor imagery, to (mostly visual) evoked potentials.

A non-invasive BCI system based on visual P300 (hereafter P300-BCI) was first proposed by Farwell and Donchin (1988) and their P300-speller is the standard reference for P300-BCIs. A “Farwell and Donchin” P300-speller enables word-spelling by flashing rows and columns of a matrix displayed on-screen and containing both letters of the alphabet and keyboard commands (like space or backspace). Therefore, a P300-BCI is rooted in the visual oddball paradigm and exploits the modifications of brain activity that arise from paying attention to a target stimulus surrounded by non relevant ones in the speller matrix. This feature makes the paradigm eligible not only for standard alphanumeric spelling; since the seminal work of Farwell and Donchin (1988) many different applications have been proposed. The feasibility of using non-character matrices of stimuli has been confirmed in studies involving healthy as well as disabled subjects spelling with different alphabets like hiragana (Ikegami et al., 2014) or logograms, i.e., Chinese alphabet (Minett et al., 2012); operating both virtual and real smart-home environments (Bayliss, 2003; Holzner et al., 2009; Carabalona et al., 2012) or browsing the Internet (Mugler et al., 2010).

For any BCI system to be used in real world, and not only in research settings, a key goal is to perform at a satisfying and reliable level, especially for disabled users (Huggins et al., 2011, 2015); with respect to P300-spellers this means efficiently maximize the difference (or *class separation*) in brain activity related to the attended stimulus (target) and ignored stimuli (non-target). To this end it is important to acknowledge the interplay between the usability of the interface and the accuracy of the classifier embedded in the BCI system (i.e., the algorithm used to classify brain activity data). Many studies have focused on the optimization of the interface parameters for a character P300–speller: matrix size (Allison and Pineda, 2003; Sellers et al., 2006) and symbols arrangements (Salvaris and Sepulveda, 2009; Li et al., 2010; Pires et al., 2012); color contrasts (Salvaris and Sepulveda, 2009; Ikegami et al., 2012); timing for stimulus presentation (Sellers et al., 2006; McFarland et al., 2011) and flash patterns (Allison and Pineda, 2006; Guger et al., 2009; Townsend et al., 2010; Ikegami et al., 2014). The leitmotiv in the proposed approaches is the evidence that no parameter combination for the stimulation matrix is optimal for all subjects in terms of achieved accuracy. Moreover, it is important to consider usability issues alongside the goal of eliciting separable brain potentials for target and non target stimuli. This means that the interface would be ergonomic, reliable, and, at the same time, fast enough to support an efficient selection. Results about BCI setting parameters on one side, and accuracy, bit rate, and users' preferences on the other, suggest that better classification results are obtained at the cost of less usable interfaces. For instance, in McFarland et al. (2011) faster flashing rates (i.e., faster interface) have a detrimental effect on accuracy (i.e., less reliable interface). Furthermore, this relation can be less apparent: if we consider also the size of the speller matrix as in Sellers et al. (2006), the reported interaction between matrix size and inter stimulus interval favors faster and smaller matrices, but this implies more steps for the user to make a final selection.

The research effort toward the best class separation led also to the acknowledgement that the performance of the classifier embedded in the BCI-speller is consistently based on the brain activity at parieto-occipital sites (Krusienski et al., 2006, 2008; Ikegami et al., 2012; Takano et al., 2014; Speier et al., 2015), notwithstanding the centro-parietal scalp distribution of the P300 peaks (Sutton et al., 1965). Moreover, some researchers took the hint of Farwell and Donchin (1988) and explicitly considered different features alongside the classical P300 (Allison and Pineda, 2003, 2006; Shishkin et al., 2009; Bianchi et al., 2010; McFarland et al., 2011). Their research on P300-spellers based on character matrices shows that attentional modulation of early event-related potential (ERP) components, like N1 and N2, results in features useful for the classifier, although, according to Shishkin et al. (2009), no effect due to physical characteristic of the character stimuli in the speller matrix was observed. Attempts to reduce performance variability across subjects and BCI-illiteracy (i.e., inefficient and unsatisfactory BCI usage) led evolve BCI-spellers from the canonical flashing approach to new paradigms where the oddball effect is obtained changing the status of speller items in other ways. Thus, despite the fact that BCI-spellers can still be referred as P300-spellers due to historical reasons, they are now also named ERP-spellers emphasizing that target and non-target stimuli are distinguished and classified by exploiting modulations of both early and late ERP components elicited by superimposing faces (either famous or dummy) on/or through movement of/items in the speller matrix (Kaufmann et al., 2011; Jin et al., 2012; Kaufmann et al., 2013; Chen et al., 2015). Results show both the feasibility of these new paradigms for character-spellers, and the improvement in classifier accuracy also for disabled users (Kaufmann et al., 2013) otherwise experiencing unsatisfactory use of the classical character flashing approach.

A diverse and hitherto quite unaddressed kind of BCI-illiteracy in using BCI-spellers becomes apparent if we consider the same group of end-users operating spellers aimed at applications different from word spelling, such as leisure activities, or interaction with a smart environment. Münßinger et al. (2010) and Zickler et al. (2013) tested different versions of Brain Painting, a BCI application for painting, both on healthy and disabled users and compared the performances with those obtained with a classical character-speller. Münßinger et al. (2010) report about two experiments, the first involving both healthy and disabled, and the second one only healthy end-users (new group). The same character-speller and two different versions of the Brain Painting were used for the two experiments. Results show a clear within-subjects variability in using both spellers, with a statistically significant drop in accuracy with respect to the character-speller in the first experiment on healthy participants using the Brain Painting. Results of the first experiment led to the new version of the Brain Painting used in the second experiment, for which there was also a drop in the Brain Painting accuracy although not statistically significant. Experimental evidence for disabled participants (3 for Münßinger et al., 2010 and 4 for Zickler et al., 2013) using the same character-speller and the two different Brain Painting versions suggests also for these end-users a drop in accuracy with respect to the character-speller, although the samples are too small and only a non inferential statistical approach is possible. Carabalona et al. (2012) proposed an icon-speller aimed at operating a real smart-home environment. This was contrasted with a character-speller for standard word-spelling in a within-subjects study with participants suffering from neurodegenerative diseases. All subjects experienced both speller types according to a randomized experimental sequence, resulting in a statistically significant lower on-line classification accuracy for the icon-speller. Thus, experimental evidence resulting from within-subjects studies suggests that the drop in accuracy is less likely related to fatigue and habituation effects, given the randomization of the experimental sequence. Results further highlight the need to elaborate on the relation between speller items and information embedded in the data submitted to the classifier. Since the classifier receives data originating from a visual cognition task, understanding how different items can shape end-users experience with BCI-spellers means looking deeper into their visual cognition and categorization processes.

Recent findings demonstrate that object and scenario categorization may be viewed as an automatic and obligatory process (Greene and Fei-Fei, 2014). Thus, when we are presented with a visual item as in the case of BCI-spellers, we automatically categorize it. In line with the terminology related to the theory developed by Rosch et al. (1976), an item can be categorized according to three possible levels of abstraction: superordinate, basic, and subordinate. For instance, a picture of a dog would be categorized at the basic level as *a dog*, but it could also be identified as *an animal* (superordinate level, more general) or as, let's say, *a Labrador* (subordinate level, less general). Jolicoeur et al. (1984) defined “entry-level” as the fastest level of categorization and argued that for atypical (with respect to the basic level) members in a category the entry-level is likely to be at the subordinate rather than at the basic level. A classic example is the penguin: as a first step it will be more likely categorized as *a penguin* (i.e., at the subordinate level), than as *a bird* (basic level). Later experimental results from Tanaka et al. (1999) pointed out the possibility to use ERP analysis to track object categorization processes of all three levels of object categorization. In their experiment, participants had to match pictures with category names at different level of abstraction by pressing a true/false key. Names and picture stimuli were presented on a computer screen, for 255 ms each, with no feedback. Results on posterior channels showed an enhanced negative deflection (N1) about 140 ms after stimulus onset, more pronounced for the subordinate category. Their analysis revealed no difference in latency among the three categories and an interaction with laterality, with a more marked negative deflection on the left side: they comment on their findings about N1 ERP component suggesting that the observed enhancement is related to the more perceptually demanding subordinate categorization level. Moreover, Tanaka and Curran (2001) reported both a right lateralization and an enhancement in the N1 component when subjects with real-world expertise (bird- and dog-watchers) were asked to categorize items in their domain of expertise with respect to when asked to categorize items outside their domain of expertise. Gathering experimental evidence arising from both BCI field and ERP tracking for semantic categorization, N1 ERP component seems a natural candidate for exploring end-user experience with BCI-spellers based on different stimuli categories.

The present study expands upon the research of Carabalona et al. (2012) and the aim is 2-fold. First, to evaluate whether the issues in using an icon-speller with respect to a character-speller observed by Carabalona et al. (2012) on disabled subjects also hold for the sample of healthy subjects considered in the present study. Second, to disentangle from an electrophysiological point of view the visual cognition process underlying end-user experience with character- and icon-spellers. The point here is not to compare characters to icons *per-se*, but to investigate whether and how different categories of BCI-items might influence the performance of a same user executing the same visual attentive task. Thus, we designed a within-subjects study considering two different 6×6 speller matrices: a standard character-speller and an icon-based one. Moreover, we manipulated both the stimulus color and flashing rate as two levels factors: white/green and fast/slow, respectively. As to the first goal, the use of the same white×fast combination as in Carabalona et al. (2012) was mandatory for comparison. With respect to the second goal, we considered a slow stimulation timing in order to avoid the ERP distortion effects (Martens et al., 2009) arising in the fast rate stimulation condition. Finally, for both character- and icon-spellers we administered a Usability questionnaire (Carabalona et al., 2012), in order to assess the subjective counterpart in BCI use.

## 2. Materials and methods

### 2.1. Participants

Eight healthy volunteers (median age in years [min–max]: 29.5 [20–36], four females) participated in this study after giving informed consent in agreement with the Declaration of Helsinki. The research project was approved by the Scientific Board of Fondazione Don Gnocchi. Eligible participants, colleagues and students rotating at the Department, were enrolled after giving their consent. The consent procedure was in oral form. Eligible participants were informed about both the aim of the research and the whole protocol (administration of paper and pencil neuropsychological tests and questionnaires; EEG acquisition with standard wet electrodes and commercial instrumentation; experimental session duration). They had the possibility to ask specific questions about the experimental data acquisition procedure and the BCI system before giving consent. It was clearly stated that participation was voluntary and that participants had the possibility to withdraw at any point of the experimental session without consequences. They were also informed about anonymous data storage, only accessible by the researcher. Data were collected according to best practice guidelines and stored anonymously. Vision was normal or corrected to normal, all subjects had a basic familiarity with computers and none of them had previous experience with BCI. Demographical characteristics are reported in Table 1.

**Table 1:** Demographic characteristics of study participants.

**Group**	**Subject**	**Gender**	**Age (years)**	**Scholarity (years)**
CH – IC	S1	F	36	20
	S2	F	20	14
	S3	M	30	11
	S4	M	27	19
IC – CH	S5	F	31	21
	S6	F	29	20
	S7	M	35	13
	S8	M	28	18

### 2.2. Study design

**Figure 1:**
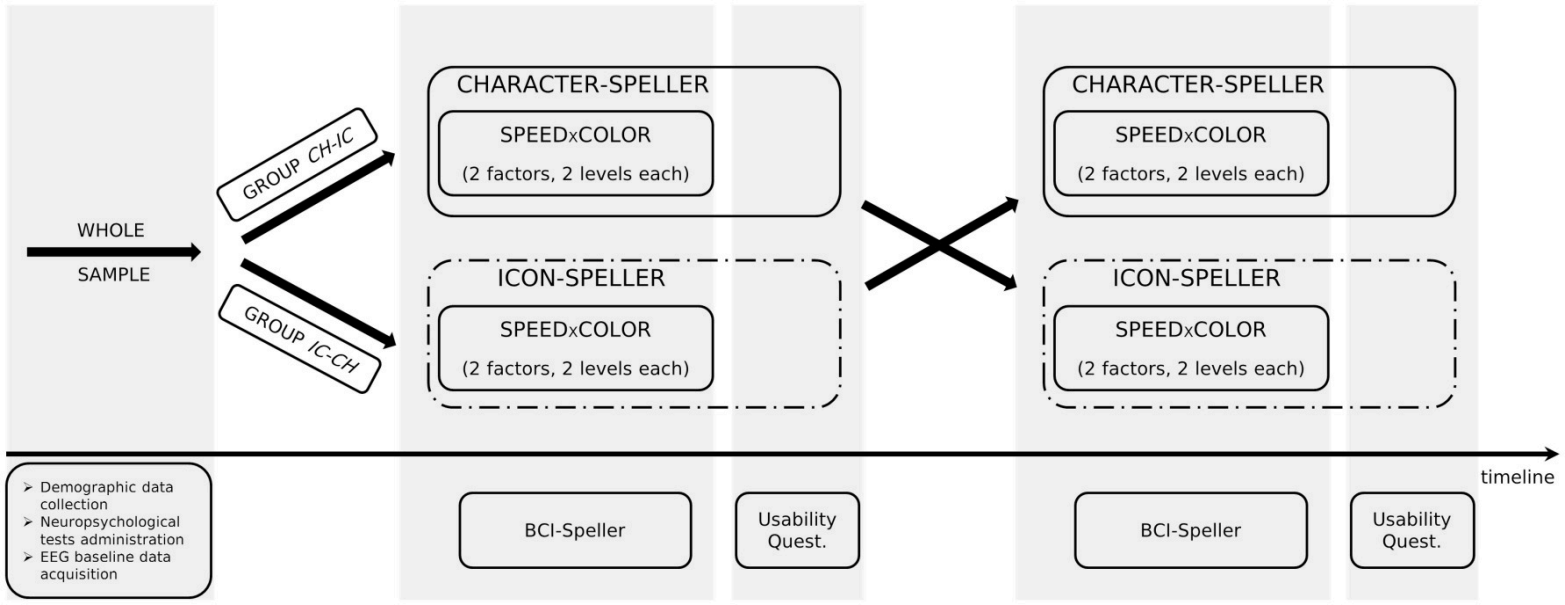
Experimental design.

In order to investigate the differences in P300-BCI performances and to allow the comparison with results reported in Carabalona et al. (2012), we considered 3 factors, 2 levels each: stimulus type (STIM: character, labeled as CH; icon, labeled as IC), stimulus color (COLOR: white, green), stimulation timing (SPEED: slow, fast). In case of slow stimulation timing (i.e., SPEED = slow), flash time was designed to be 100 ms and dark time between two flashes to be 900 ms. For fast stimulation timing (i.e., SPEED = fast), flash time was designed to be 60 ms and dark time between two flashes to be 10 ms. Moreover, since we were also interested in the subjective evaluation of the usability of each speller type *in itself*, we designed experimental sequences interlacing a cross-over approach with a factorial one, as depicted in Figure 1. Given the stimulus type (CH or IC), order and sequence effects for the SPEED×COLOR combinations were counteracted using a balanced Latin square design. Groups corresponding to each of the two STIM sequences (i.e., *CH-IC* or *IC-CH*) were matched for gender and age. Speller types used for BCI acquisitions were always 6×6 matrices of stimuli on black background, whereas flashing symbols flashed from dark gray to white or green, according to the level of factor COLOR.

### 2.3. Experimental set-up

All data acquisitions were performed in the smart home **DAT**-**D**omotic, **A**ssistive technologies permanent exhibition, and occupational **T**herapy (Andrich et al., 2006), a facility built in the Santa Maria Nascente Care and Research Institute of Don Gnocchi Foundation, in Milan, Italy. The BCI system was integrated into the EIB/Konnex environmental control system of DAT using a configurable network interface designed at the University of Parma, called **FEIM**-**F**ield **E**thernet **I**nterface **M**odule (Grossi et al., 2007).

Since the P300-BCI is based on a paradigm requiring visuo-spatial attention, participants underwent a neuropsychological evaluation before instrumentation for BCI. End-users' performance and experience with respect to character- and icon-spellers were assessed using on-line selection accuracy of the specific classifier as well as off-line biosignal analysis of EEG data acquired during rest and BCI sessions. Details for each assessment procedure are given below.

#### 2.3.1. Neuropsychological evaluation

We evaluated participants' working memory as well as selective attention and scan velocity administering three different neuropsychological tests. Since we aimed at assessing the comparability with the results reported in Carabalona et al. (2012), we used the same tests, namely: Mini Mental State version of the Serial Seven Subtraction Test (SSST) (Folstein et al., 1975) and the Attentive Matrices Test (AMT) (Spinnler and Tognoni, 1975) as in Carabalona et al. (2012). Moreover, we added the Bells Cancellation Test (Gauthier et al., 1989) in order to assess selective attention and scan velocity specifically for icons.

#### 2.3.2. Biosignal acquisition

During biosignal acquisition, subjects sat in a comfortable chair approximately 60 cm in front of a 50.8 cm (20-inch) LCD-screen (refresh rate: 60 Hz). Tilt, brightness and contrast of the computer monitor were adjusted in order to guarantee comfort and clear vision. Signals were digitized at 24 bits and sampled at 256 Hz by means of a single biosignal amplifier (gUSBamp, g.tec). Impedances were maintained below 10 kΩ. A 50 Hz notch filter was used and data were also bandpass filtered between 0.5 and 30 Hz. For EEG acquisition, we used eight electrodes according to the extended International 10–20 system (Nuwer et al., 1998): F_*z*_, C_*z*_, P_3_, P_*z*_, P_4_, PO_7_, O_*z*_, and PO_8_. This EEG channels set is the same used in Carabalona et al. (2012). Data on eye movements (EOG) were derived from four electrodes: two placed above and below the left eye, two placed about 1-cm from the external canthus of each eye. As ground and reference, we selected Fp_*z*_ and right mastoid, respectively.

#### 2.3.3. P300-BCI data acquisition protocol

**Figure 2:**
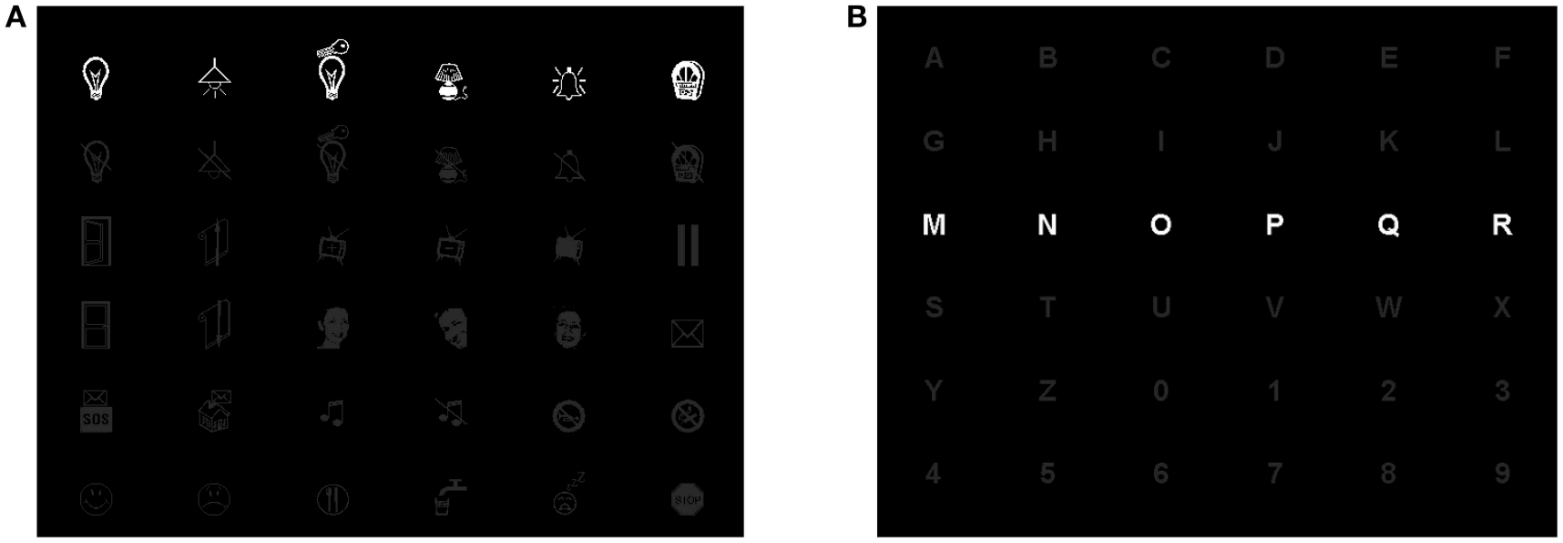
Screenshots of 6x6 matrices used for the white icon–speller **(A)** and the white character–speller **(B)**.

For each STIM×COLOR×SPEED combination in the assigned experimental sequence, participants performed a P300-BCI session consisting of two phases: training and performance. Both were in copy spelling, thus the subject had to spell a predefined sequence of symbols. For each session, a 6 × 6 matrix containing only alphanumeric characters or only icons (one in each cell, see Figure 2) was presented to the subject and Fisher's Linear Discriminant Analysis (hereafter LDA, the algorithm is detailed in subsection 2.5.1) was used as classifier as in Carabalona et al. (2012). During the training phase, the subject communicated a predefined meaningful word (or string of icons) and, since the classifier was still in its “learning” phase, the BCI system gave a feedback to the user printing on the screen the symbol “@” at the end of of every flashing sequence and no action was executed in the smart home. This type of feedback was labeled as *meaningless* because the printed symbol was not included in the character- nor in the icon-speller matrices. The whole data-set was then used off-line by the BCI system to initialize the classification algorithm. During the performance phase, the subject had to communicate a different predefined meaningful word (or string of icons) and the epoched data-set related to each spelled symbol was classified at the end of the flashing sequence according to the classifier learned off-line after the training phase. In this case the subject received a meaningful feedback at the end of each series of random flashes, as a result of an on-line classification. This means that, depending on the speller type, one of the two events may occur: either a character is printed on the screen, for the character-speller, or an icon is printed on the screen followed by the execution of the corresponding action in the smart home, for the icon-speller. After each BCI feedback, there was a pause lasting 4 seconds which allowed participants time to relax and to locate the next target. Subjects were fully aware that the training phase produced meaningless feedback as well as that the LDA algorithm used data acquired during the training phase in order to extract and learn how to classify the features elicited by the cognitive task: they had been told in advance to ignore the symbol printed on the screen at the end of every flashing sequence in the training phase.

### 2.4. Experimental schedule

Before subject instrumentation, demographic data were acquired by means of a brief interview. Subsequently, Mini Mental State version of the Serial Seven Subtraction Test (SSST), Attentive Matrices Test (AMT) and Bells Cancelation Test were administered. After neuropsychological assessment, subjects received a sort of BCI manual. This was a booklet describing each stage of BCI use (training phase and meaningless feedback, off-line data elaboration, performance phase with meaningful feedback) by means of screenshots with suitable detailed captions, one for each page. Subjects were given all the needed time to read the booklet and the possibility to ask questions during or after reading. Then, subjects were instrumented and basal data were acquired at rest (eyes open, without cognitive stimulation). The different P300-spellers were subsequently administered according to the assigned experimental sequence.

Before each P300 session, subjects could visually explore a still (i.e., not flashing) speller matrix depicted on the computer monitor, without any time pressure. This was intended to give the subjects the possibility to familiarize with the stimulus matrix in terms of both symbol type and arrangement within the matrix itself. Participants were also allowed to ask questions about the meaning of icons. At the very beginning of each experimental sequence, i.e., the very first time a participant was presented with the speller matrix, time spent for scanning the still matrix was registered.

Participants had to spell 5-characters meaningful words or 5-icons strings. The word (string) to spell for the training phase was different from that spelled during the performance phase. In sum, we used a fixed set (with respect to participants) of eight different 5-characters meaningful words and of eight different 5-icons strings. A different LDA classifier was learned for each training-performance pair. According to study design, subjects performed both whole character-based BCI and icon-based BCI experimental sequences. After completing each assigned sequence, they had the possibility to rest and to express their opinion on their BCI experience. They received a Usability questionnaire (Carabalona et al., 2012) pertaining to four domains: Satisfaction, Ease of Use, Usefulness and Ease of Learning. Overall, participants rated fourteen statements on seven-point Likert scales ranging from “strongly disagree” (labeled with 1) to “strongly agree” (labeled with 7).

### 2.5. EEG data analysis

Since the present study has two different aims, we performed diverse analyses on acquired EEG data.

First, we aimed at evaluating whether the within-subjects BCI illiteracy observed by Carabalona et al. (2012) on disabled subjects using both icon- and character-speller also hold for the sample of healthy subjects considered in the present study. Thus, the goal of the EEG data analysis here is to compute the classifier accuracy for each speller type during the performance phase (i.e., on-line) in order to assess whether the same issue is present. This is detailed in subsection 2.5.1.

Our second aim was to disentangle from an electrophysiological point of view the visual cognition process underlying the end-user experience with character- and icon-spellers. Therefore, we performed off-line analyses in order to investigate whether and how different categories of BCI-items might influence the performance of a same end-user executing the same visual attentive task. The analyses are detailed in subsection 2.5.2.

#### 2.5.1. Computation of classifier accuracy

According to the first aim, acquired brain activity was submitted to the LDA classifier embedded in the BCI system as in Carabalona et al. (2012). EEG data are down-sampled to 64 Hz, epoched using with a window length of 800 ms (100 ms before and 700 ms after stimulus onset), baseline corrected using pre-stimulus data, and filtered with a moving average filter (3 samples). For each trial (i.e., symbol to spell) rows and columns of the matrix flashed 15 times each and flashing order of rows and columns was randomly selected. As regards the training data-set, this led to 150 target data (15 flashes × 2 [one row and one column] × 5 [number of symbols spelled]) and to 750 non-target data (15 flashes × 10 [5 rows and 5 columns] × 5 [number of symbols spelled]). Thus, the training data matrix submitted to the classifier consisted of 900 rows (i.e., 150 target+750 non-target) and 120 columns (15 time samples × 8 [number of electrodes]). With respect to the performance phase, at the end of each trial (i.e., at the end of the flashing sequence for each symbol to spell) the learned classifier was used to assign a weight to each row and column. The weight are computed as cumulative sum of posterior probabilities for target-class membership. The algorithm identifies the row *r* and the column *c* with highest cumulative posterior probability and selects the corresponding element, i.e., the one indexed with (*r, c*), in the speller-matrix.

#### 2.5.2. Electrophysiological analysis of end-user experience

In line with the second aim, continuous EEG was first preprocessed in order to remove ocular artifacts with an adaptive procedure based on concurrent EOG data. Then, consistently with the approach used during BCI sessions, EEG data were windowed into epochs (100 ms pre-stimulus to 700 ms post-stimulus onset) and baseline corrected from 100 ms pre-stimulus to stimulus onset. ERPs were calculated for each participant and each experimental condition for every electrode in the EEG-set. We considered **SPEED** = **slow** and **SPEED** = **fast** separately because fast stimulation timing dramatically alters the ERP morphology hence making very difficult to track potential visual categorization effects.

ERP data for **SPEED** = **slow** were analyzed using time-domain methods, with reference to approach and results described in Tanaka et al. (1999) and in Tanaka and Curran (2001) with respect to ERP tracking of semantic categorization process. We identified two ERP peaks after stimulus onset: the local maximal negative deflection (N1) in the 130–200 ms time window, and the local maximal positive deflection (P300) in the 250–450 ms time window. We evaluated both training and performance phases. First, we aimed at assessing the consistency with results reported in Tanaka et al. (1999), where participants did not receive any feedback and a greater (i.e., more negative) N1 component was associated with categorization at subordinate level with respect to categorization at the basic level for posterior sites. Thus, we assessed data from training phase (meaningless feedback and no action in the smart home) considering both N1 and P300 for the whole electrode set. The experimental evidence in Tanaka et al. (1999) about greater negative deflection in N1 for subordinate level of semantic categorization led to formulation of one-sided alternative hypotheses. Moreover, only for C_*z*_, we added three time windows (50 ms each) ranging from 200 to 350 ms after stimulus onset. These three subsets were then averaged in each time window for statistical analysis.

Subsequently, we aimed at evaluate the consistency with results reported in Tanaka and Curran (2001) about the relation between real-world expertise and semantic categorization level on a sample of real-world experts. They asked bird- and dog-watchers to perform semantic categorization with respect to stimuli both outside and in their domain of expertise: according to their results, real-world experts showed a right lateralization for the N1 component, with a more negative N1 when the stimulus was in their domain of expertise as when it was outside. As regards BCI experience, we can consider participants as real-world experts with respect to characters and novices with respect to icons. Moreover, in contrast with the training phase, the presence of a meaningful feedback allows the end-users to really interact with the environment, making the use of the BCI-speller less detached from reality than it was during the training. We thus regarded the performance phase as the “real-world expertise” phase and analyzed N1 only for PO_7_ and PO_8_. Also in this case the greater negative deflection in N1 reported in Tanaka and Curran (2001) for not-novel stimuli (which are the items in the character-speller for the present study) led to formulation of one-sided alternative hypotheses. Finally, we considered the P300 extracted from all electrodes, in order to assess potential differences also in this ERP component.

For the case **SPEED** = **fast**, we analyzed only epoched data related to the performance phase for each STIM×COLOR combination and posterior electrodes: since the fast stimulation dramatically modifies ERP waveforms in the time domain, data were analyzed by means of time-frequency decomposition, in order to enhance both effects related to stimulus-locking and the steady state visual evoked potentials (SSVEP) induced by non-target stimuli. According to the approach described in Tallon-Baudry et al. (1997), ERP data were convolved with Morlet's wavelets *w*(*t, f*_0_) defined as:

(1) $$\begin{array}{r} w\left(t,f_{0}\right)=A\;\exp\left(-t^{2}/2\sigma_{t}^{2}\right)\;\exp\left(2i\pi f_{0}t\right) \end{array}$$

Each wavelet has a Gaussian shape characterized in time and frequency domain by σ_*t*_ and σ_*f*_ respectively, with σ_*f*_ = 1/2πσ_*t*_, and a normalization factor $A={\left(\sigma_{t}\sqrt{\pi }\right)}^{-1/2}$. Moreover, the family is characterized by the constant ratio *k* = *f*_0_/σ_*f*_ that should be equal to (or greater than) 5. For our data analysis we chose *k* = 7, with *f*_0_ ranging from 5 to 20 Hz, in 1 Hz steps. This leads, for *f*_0_ = 5 Hz to 2σ_*f*_ = 1.43 Hz and 2σ_*t*_ = 445.63 ms and for *f*_0_ = 20 Hz to 2σ_*f*_ = 5.71 Hz and 2σ_*t*_ = 111.41 ms.

For each trial we compute the normalized complex time-varying energy for the signal *s*_*j*_(*t*) in trial j, with j = 1…N:

(2) $$\begin{array}{r} P_{j}\left(t,f_{0}\right)=w\left(t,f_{0}\right)\times s_{j}\left(t\right)/|w\left(t,f_{0}\right)\times s_{j}\left(t\right)| \end{array}$$

Finally, as in Tallon-Baudry et al. (1997), we computed the phase-locking factor (PLF). This index is stimulus-locked and, independently of signal amplitude, indicates the consistency of phase values across trials at different frequencies: it ranges from 0 (no phase synchronization across trials) to 1 (perfectly phase-locked activity).

PLF is expressed as the modulus of the averaged *P*_*j*_(*t, f*_0_) across N trials:

(3) $$\begin{array}{r} PLF\left(t,f_{0}\right)=|\frac{\Sigma_{j}\left(P_{j}\left(t,f_{0}\right)\right)}{N}| \end{array}$$

We computed PLF both for correct and wrong selections, separating target and non-target data. For instance, for a subject with 3 correct selections in a sequence of 5, *N_TARGET_correct__* = 3*30 = (number of correct selections)*(number of target stimuli) and *N_TARGET_wrong__* = 2*30 = (number of wrong selections)*(number of target stimuli); *N_NON-TARGET_correct__* = 3*150 = (number of correct selections)*(number of non-target stimuli) and *N_NON-TARGET_wrong__* = 2*150 = (number of wrong selections)* (number of non-target stimuli).

Since the PLF is a function of both time and frequency, for each subject we collapsed PLF data along the time dimension using the median value. Each subject is then represented with a vector of 16 (the frequency span used for PLF calculation) median values for each STIM×COLOR combination. Two data-set related to correctly performed trials were then created grouping median PLF values for correct target selections: one for target and one for non-target data. The two data-set associated with correct performances were then considered as reference set for median PLF values related to wrong selection trials.

#### 2.5.3. Influence of the information embedded the N1 component on classifier selection accuracy

As a last step, since the scalp distribution of the N1 component involves parieto-occipital electrodes, we excluded P_*z*_, PO_7_, and PO_8_ channels data from the original training and performance phase data-set in order to assess the direct influence of the information embedded in the N1 component on classifier selection accuracy. We performed off-line analyses on EEG data from the reduced data-set for every STIM×COLOR×SPEED combination and for each subject. Each reduced training data-set was submitted to the LDA classifier as described in subsection 2.5.1. The newly trained classifier was then used to classify each reduced data-set corresponding to the performance phase. Thus, we computed new classification weights (i.e., cumulative sums of posterior probabilities of target class membership) and subsequent selection accuracy for each target.

### 2.6. Statistical analysis

All analyses were performed using R (R Core Team, 2014). With respect to descriptive statistics (i.e., grand average, median, maximum, relative frequency, as well as Spearman's correlation coefficient) computations were performed using standard functions implemented in R (package *stats*). Plots were obtained using the package *ggplot2* (Wickham, 2009) and the function “balloonplot” implemented in the package *gplots* (Warnes et al., 2016). With respect to the analysis performed on the N1 and P300 components for factor SPEED = slow, considering the Latin-square design and after a preliminary inspection of ERP data we assumed a negligible period effect in testing differences in character- and icon-spellers. This means that we considered data as they had been collected using a within-Ss factorial design with the two factors STIM (2 levels: character, CH and icon, IC) and COLOR (2 levels: white, WH and green, GR). We therefore performed a permutational analysis of variance for repeated measures using the function “ezPerm” implemented in the package *ez* (Lawrence, 2013). Effect sizes are computed using the function “ezANOVA” implemented in the same package (Lawrence, 2013) and referencing to Bakeman (2005). One-way analysis of variance for repeated measures was performed using Friedman test (function “friedman.test” in package *stats*). For paired data comparisons, Wilcoxon Rank Sum (function “wilcox.test” in package *stats*) or sign test (function “signmedian.test” in package *signmedian.test*, Yu and Yang, 2015) were chosen depending on the asymmetry for distributions of differences. As index for effect size we used in this case Cliff's delta according the approach implemented in the package *effsize* (function “cliff.delta”, Torchiano, 2016). Statistical significance was set at 0.05. In case of multiple testing, family-wise error rate was controlled according to the Holm approach (*holm* option of the “p.adjust” function of the R standard package *stats*).

## 3. Results

In order to evaluate cognitive differences in using a character-speller with respect to an icon-based one, we considered many aspects. The following subsections detail the results obtained from various assessments procedures and EEG data analyses.

### 3.1. Neuropsychological assessment

Scan times for AMT were (median [25–75%]): 18.3 [15.2–22.9], 23.8 [21.9–26.9], and 30.1 [27.0–31.1] seconds for the three matrices, respectively. Friedman test was significant (*P*-value = 0.0046) and *post hoc* test confirmed that scant time increased with task difficulty. Results were (Wilcoxon Rank Sum test, one-sided alternative hypothesis with Holm correction): scan time AMT2 vs. AMT1 Cliff's delta = 0.5313, *P*-Value = 0.055 (uncorrected 0.055); scan time AMT3 vs. AMT2 Cliff's delta = 0.5, *P*-Value = 0.0117 (uncorrected 0.0039) and scan time AMT3 vs. AMT1 Cliff's delta = 0.7813, *P*-Value = 0.0156 (uncorrected 0.0078). The score (i.e., the number of correct subtractions in the five trials) in the SSST was quite uniformly distributed in the two groups, being 5,3,5,4 for the CH-IC group and 5,2,4,5 for the IC-CH group. Only one subject performed critically in the Bells Test (S2, 11 missing targets: 4 in the left and 5 in the right side), being also the worst performer in the last attentive matrix (7 missing targets) and always the one with the minimum scan time (44.4 and 20.5 s, in Bells Test and third attentive matrix respectively).

### 3.2. Other behavioral data

Time data related to the possibility to familiarize with speller matrices refer to time to scan *still*, not flashing, speller matrices at the very beginning of each experimental sequence. This was 15.5 s [12.9–22.0] for the character-speller, and 41.5 s [34.2–70.4] for the icon-speller. Scan time for the icon-speller matrix was longer than scan time for the character-speller matrix, with a statistically significant *P*-value of 0.0352 (sign test with one sided alternative hypothesis, Cliff's delta = 0.9063). Relations (Spearman's ρ) between data from neuropsychological assessment (AMT and Bells Test) and scan time for still speller matrices (0.476, 0.691, 0.595, 0.024 for character-speller; 0.333, 0.405, 0.309, 0.595 for icon-speller) show weak direct correlations, although not statistically significant.

### 3.3. BCI performances

**Figure 3:**
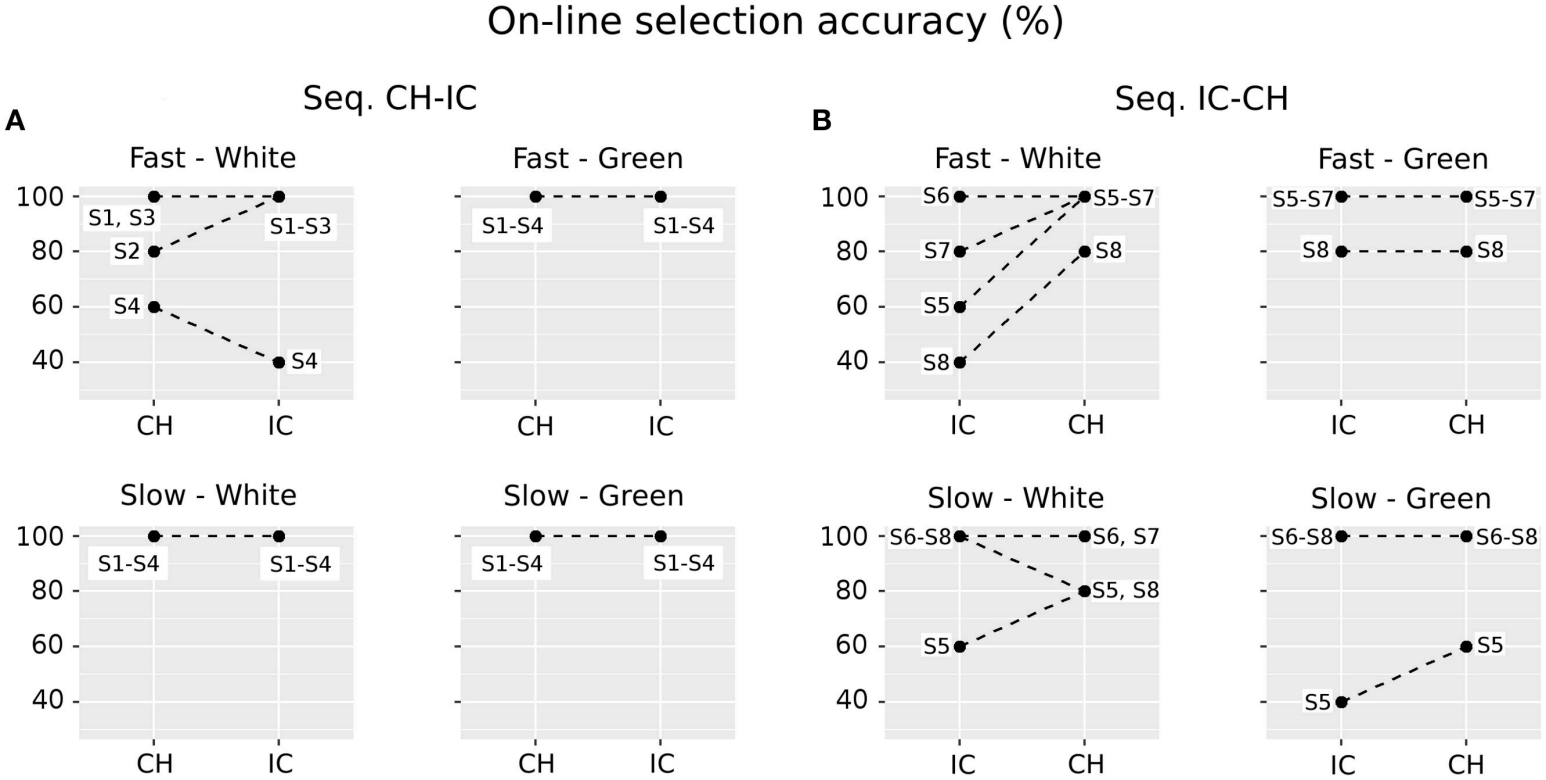
Paired data plots referring to performances with the different spellers for each subject, explicitly labeled with letter S and consecutive numbers. Since data for selection accuracy can overlap, a single dot can correspond to more than one subject. Therefore each dot is labeled: a label with dash (as in *S1–S3*) means S1, S2, and S3; a label with comma (as in *S1, S3*) means S1 and S3. Results are reported in panels **(A)** and **(B)** according to the assigned experimental group (Seq. *CH-IC* or *IC-CH*, i.e., first character-speller and then icon-speller or the other way around) timeline. Dots are linked with dashed lines in order to highlight the within-subjects variability in performing with the various spellers. As regards each subplot: White and Green labels refer to the stimulus COLOR (factor COLOR, two levels), whereas Fast and Slow labels refer to stimulus timing (Factor SPEED, two levels). Stimulus type: CH, character-speller; IC, icon-speller.

On-line classification accuracy in the different experimental combinations of STIM (CH, IC), COLOR (white, green) and SPEED (fast, slow) is depicted in Figure 3. The achieved on-line accuracy is plotted using dots: since many subjects can perform at the same level, a single dot could also correspond to more than one subject. Therefore each dot is labeled: a label with dash (as in *S1–S3*) means S1, S2, and S3; a label with comma (as in *S1, S3*) means S1 and S3. Data are also plotted in panels A and B according to the experimental sequence the participant was assigned to, i.e., *CH-IC* or *IC-CH*, as in Figure 1. Finally, dots are linked with dashed lines in order to highlight the within-subjects variability in performing with the various spellers. Only three subjects reached 100% accuracy in all experimental conditions (S1, S3, S6), whereas worst performances are 40% (S4 and S8, IC×white×fast; S5, IC×green×slow) and 60% (S4, CH×white×fast; S5, IC×white×fast, CH×green×slow and IC×white×slow). The combination IC×white×fast emerges as the more heterogeneous one among subjects, although classification accuracy for white×fast icons was not significantly worse from that for white×fast characters (sign test with one-sided alternative hypothesis, *P*-value = 0.1875).

### 3.4. Electrophysiological analysis of end-user experience

According to the approach described in subsection 2.5.2, results reported in the following subsections are stratified with respect to the two levels of the factor SPEED, which refers to the stimulation timing used for the P300-spellers. Slow stimulation timing (flash time = 100 ms, dark time = 900 ms) removes the distortion of ERP waveforms; fast stimulation timing (flash time = 60 ms, dark time = 10 ms) refers to a feasible P300-BCI application, since it enables fast communication and interaction.

#### 3.4.1. Off-line ERP analysis − SPEED = slow

**Figure 4:**
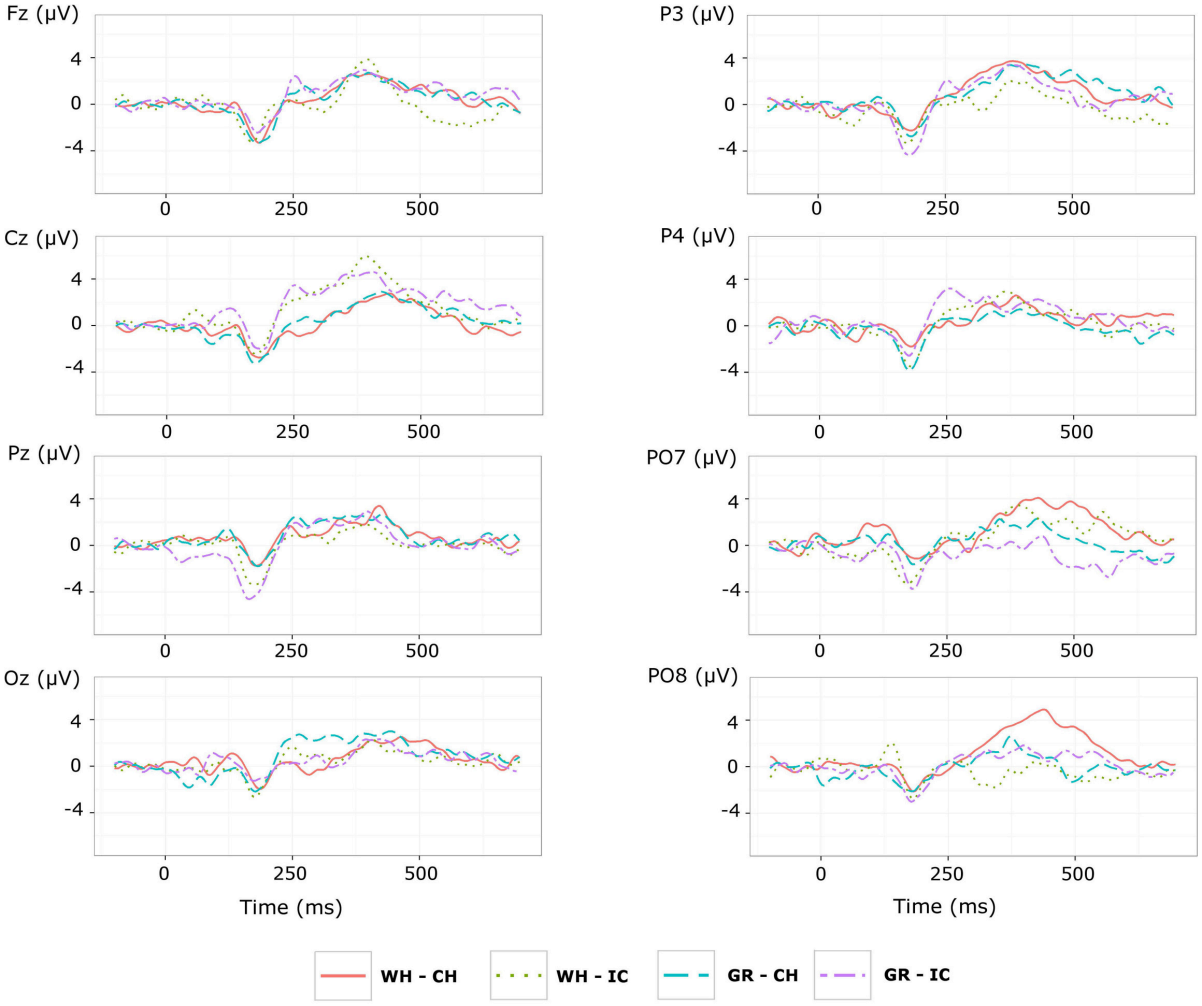
Target ERPs in BCI-set (F_*z*_, C_*z*_, P_3_, P_*z*_, P_4_, PO_7_, O_*z*_, and PO_8_) for the *training phase* and slow stimulation timing (flash time = 100 ms, dark time = 900 ms). Data are epoched (from 100 before to 700 ms after stimulus onset) and baseline corrected for each-speller type. WH-CH, white character-speller; WH-IC, white icon-speller; GR-CH, green character-speller; GR-IC, green icon-speller.

Grand average target-ERPs relative to the **training phase** for slow stimulation timing in each STIM×COLOR combination are reported in Figure 4 for the different conditions. Data for N1 component are reported in Table 2 and corresponding effect sizes for each electrode and STIM×COLOR combination are reported in Table 3. With respect to the P300, the peak amplitudes [μV] (latencies, [ms]) at C_*z*_ were: 2.781 (439.063) and 2.917 (423.438) for white and green character-speller; 5.999 (392.188) and 4.612 (407.813) for white and green icon-speller.

**Table 2:** Speed = SLOW: Amplitude (μV) and latency (ms) for N1 component during training phase.

	**F_*z*_**		**C_*z*_**		**P_*z*_**		**O_*z*_**	
	**Amp**	**Lat**	**Amp**	**Lat**	**Amp**	**Lat**	**Amp**	**Lat**
WH-CH	−3.309	185.156	−2.742	181.250	−1.742	177.344	−1.938	185.156
GR-CH	−3.325	177.344	−3.253	173.438	−1.778	181.250	−2.167	177.344
WH-IC	−3.226	169.531	−2.445	177.344	−3.348	173.438	−2.656	177.344
GR-IC	−2.439	177.344	−2.014	189.063	−4.621	165.625	−1.251	173.438
	**P**_3_		**P**_4_		**PO**_7_		**PO**_8_	
	**Amp**	**Lat**	**Amp**	**Lat**	**Amp**	**Lat**	**Amp**	**Lat**
WH-CH	−2.226	181.250	−1.777	181.250	−1.119	192.969	−2.131	181.250
GR-CH	−2.732	181.250	−3.835	177.344	−1.618	185.156	−2.110	177.344
WH-IC	−3.314	173.438	−3.485	181.250	−3.269	173.438	−2.762	181.250
GR-IC	−4.332	177.344	−2.568	177.344	−3.731	185.156	−3.007	177.344

*Stimulus type: CH, character-speller; IC, icon-speller; Stimulus color: WH, white; GR, green*.

**Table 3:** Speed = SLOW: Generalized Eta-Squared measure of effect size for N1 amplitude during training (all electrodes) and performance (only for PO_7_ and PO_8_, in bold) phases.

	**F_*z*_**	**C_*z*_**	**P_*z*_**	**O_*z*_**
STIM	0.0130	0.0245	0.1305	0.0003
COLOR	0.0083	0.0001	0.0128	0.0105
STIM×COLOR	0.0090	0.0094	0.0115	0.0200
	**P**_3_	**P**_4_	**PO**_7_	**PO**_8_
STIM	0.0345	0.0018	0.0942	0.0178
COLOR	0.0113	0.0119	0.0053	0.0004
STIM×COLOR	0.0013	0.0755	0.0000	0.0005
			**PO**_7_	**PO**_8_
STIM			**0.0026**	**0.1762**
COLOR			**0.0111**	**0.0017**
STIM×COLOR			**0.0106**	**0.0272**

*STIM refers to stimulus type: CH, character-speller; IC, icon-speller; COLOR refers to stimulus color: WH, white; GR, green*.

Referring to ERPs for training phase-target condition for each electrode and STIM×COLOR combination, permutational analysis of variance for the N1 component amplitude resulted, after Holm correction for the whole electrode set (i.e., 8 electrodes), in a statistically significant STIM main effect for P_*z*_ (Holm corrected *P*-Value = 0.035; uncorrected: 0.005) and PO_7_ (Holm corrected *P*-Value = 0.032; uncorrected: 0.004). *Post hoc* analysis was then performed on ERP data for these two electrodes, collapsing data with respect to the factor COLOR. N1 component amplitude for STIM = IC was larger (i.e., more negative) than for STIM = CH for both P_*z*_ and PO_7_ (Cliff's delta = −0.4375 and −0.4063, respectively. Sign test with one sided alternative hypothesis: Holm corrected *P*-Value = 0.0078, uncorrected = 0.0039).

Permutational analysis of variance for each STIM×COLOR combination on C_*z*_ data averaged in the three time windows A (200-250 ms, generalized effect sizes: STIM = 0.1736, COLOR = 0.0017, STIM×COLOR = 0.0005), B (250–300 ms, generalized effect sizes: STIM = 0.2395, COLOR = 0.0218, STIM×COLOR = 0.0065) and C (300–350 ms, generalized effect sizes: STIM = 0.17, COLOR = 0, STIM×COLOR = 0) led to the following Holm corrected (uncorrected) *P*-Values for STIM main effect : 0.052 (0.043), 0.033 (0.011), and 0.052 (0.026) for time-window A, B, and C respectively. *P*-values for STIM×COLOR interaction and COLOR effects were far from statistical significance. Given the significant result on window B, *post hoc* analysis on collapsed data with respect to the factor COLOR showed a greater amplitude for STIM = IC than for STIM = CH (Cliff's delta = 0.7188; *P*-value = 0.035, sign test with one sided alternative hypothesis). Permutational analysis of variance for the P300 component led to *P*-values far from statistical significance.

**Figure 5:**
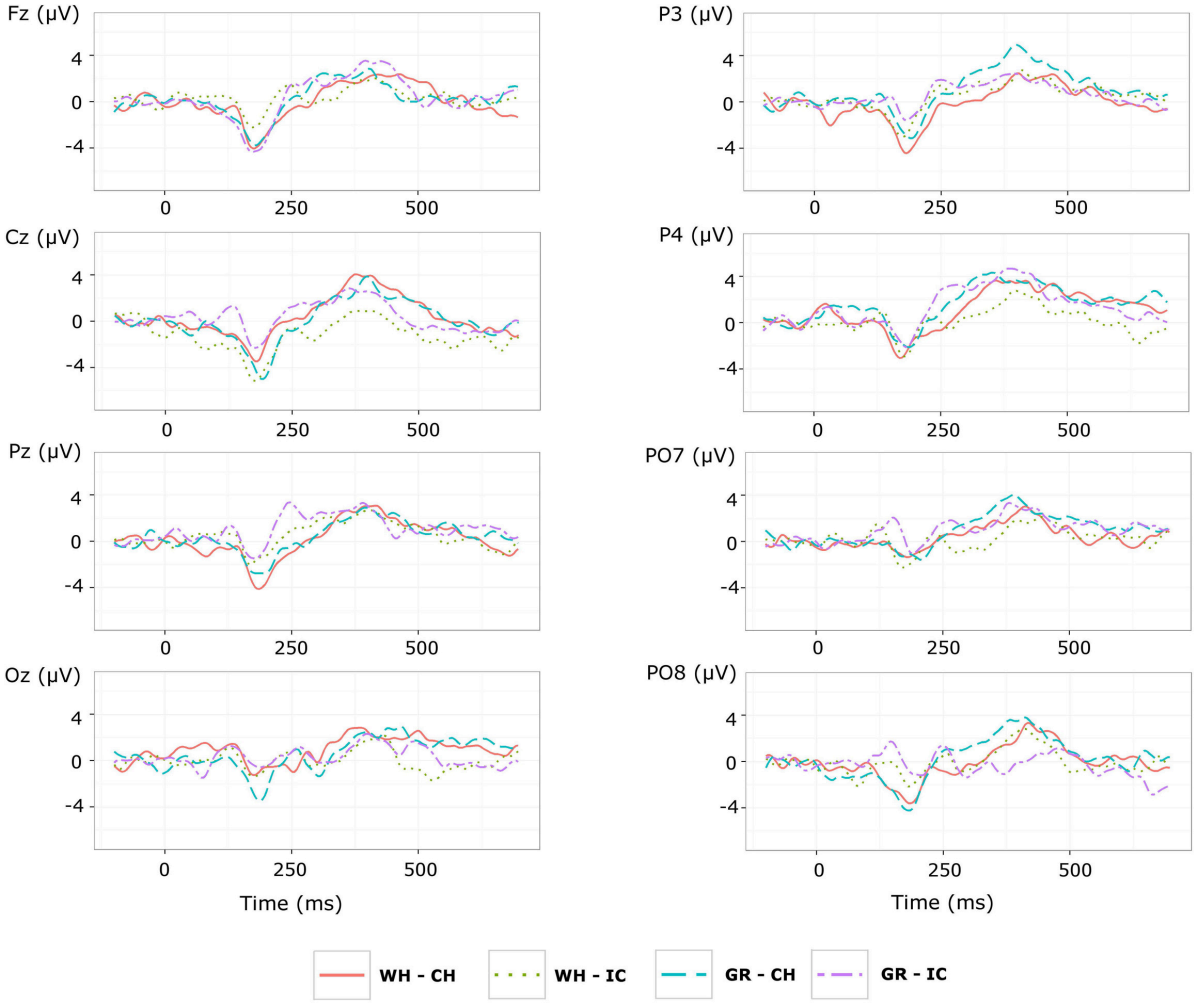
Target ERPs in BCI-set (F_*z*_, C_*z*_, P_3_, P_*z*_, P_4_, PO_7_, O_*z*_, PO_8_) for the *performance phase* and slow stimulation timing (flash time = 100 ms, dark time = 900 ms). Data are epoched (from 100 before to 700 ms after stimulus onset) and baseline corrected for each -speller type. WH-CH, white character-speller; WH-IC, white icon-speller; GR-CH, green character-speller; GR-IC, green icon-speller.

With respect to the performance phase, grand average ERPs for target and slow stimulation timing in each STIM×COLOR combination are reported in Figure 5. Data for N1 component for each electrode and STIM×COLOR combination are reported in Table 4. We considered for statistical analysis the N1 component only for parieto-occipital electrodes PO_7_ and PO_8_: corresponding effect sizes are reported in bold in Table 3. A statistically significant STIM main effect was observed on the N1 component amplitude for PO_8_ (Holm corrected *P*-Value = 0.03; uncorrected: 0.015). We consequently collapsed data for factor COLOR for PO_8_ and compared data for STIM = CH and STIM = IC: N1 component amplitude for STIM = CH was larger (i.e., more negative) than for STIM = IC (Cliff's delta = −0.5937. Sign test with one sided alternative hypothesis: 0.035). With respect to the P300 component, permutational analysis of variance led to *P*-values far from statistical significance.

**Table 4:** Speed = SLOW: Amplitude (μV) and latency (ms) for N1 component during performance phase.

	**F_*z*_**		**C_*z*_**		**P_*z*_**		**O_*z*_**	
	**Amp**	**Lat**	**Amp**	**Lat**	**Amp**	**Lat**	**Amp**	**Lat**
WH-CH	−4.037	177.344	−3.473	177.344	−4.122	185.156	−1.244	177.344
GR-CH	−3.765	177.344	−4.994	189.063	−2.763	196.875	−3.462	189.063
WH-IC	−2.213	173.438	−5.153	177.344	−2.020	169.531	−1.367	173.438
GR-IC	−4.323	173.438	−2.304	177.344	−1.472	177.344	−0.576	185.156
	**P**_3_		**P**_4_		**PO**_7_		**PO**_8_	
	**Amp**	**Lat**	**Amp**	**Lat**	**Amp**	**Lat**	**Amp**	**Lat**
WH-CH	−4.425	181.250	−3.046	169.531	−1.351	181.250	−3.610	185.156
GR-CH	−3.144	189.063	−2.115	185.156	−1.338	196.875	−4.231	181.250
WH-IC	−2.985	173.438	−2.909	177.344	−2.264	169.531	−2.148	185.156
GR-IC	−1.576	181.250	−2.108	181.250	−1.028	189.063	−1.114	196.875

*Stimulus type: CH, character-speller; IC, icon-speller; Stimulus color: WH, white; GR, green*.

#### 3.4.2. Off-line ERP analysis − SPEED = fast

**Figure 6:**
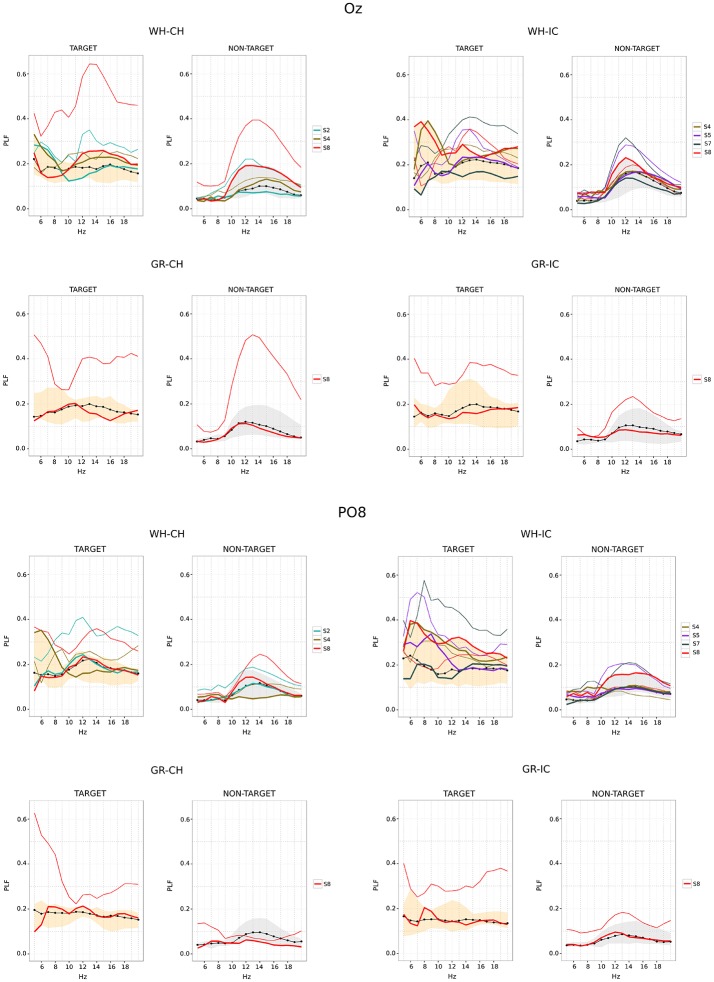
Time-frequency analysis for O_*z*_ (upper panel) and PO_8_ (lower panel) epoched data (performance phase and SPEED = fast). For each subject, Phase locking factor (PLF) is summarized along time using the median value. PLF associated with correct selections are summarized across subjects using median (black dot-line) and minimum/maximum (shaded area) of subjects' medians. Single lines (thick and thin) refers to subjects who did both correct (thick line) and wrong selections (thin line). WH-CH, white character-speller; WH-IC, white icon-speller; GR-CH, green character-speller; GR-IC, green icon-speller.

Phase locking values for O_*z*_ and PO_8_ are reported in Figure 6. For each STIM×COLOR combination plot are paired for target and non-target data. The correct performances data-set (obtained grouping the median PLF values for correct selections) is graphically summarized reporting its median (the black dot-line) and minimum and maximum (the shaded area). The SSVEP induced by flashing of non-target data is clearly recognizable in each NON-TARGET plot for O_*z*_ and the SSVEP effect seem also present in target data. In each plot it is also stressed the fact that some subjects performed bad in some trials and well in some other. This is achieved representing the median PLF values for these subjects in a 2-fold way: the bold line (which is within the shaded area) is related to the correct performance (i.e., with correct selection); the thin line, with the same color code as the thick one, is related to the bad performance (i.e., with wrong selection). Results for bad performances show greater dispersion, mostly peaking in the 10–15 frequency band.

#### 3.4.3. Influence of the information embedded the N1 component on classifier selection accuracy

Results about the selection accuracy achieved using the reduced data set, i.e., excluding P_*z*_, PO_7_, and PO_8_., are reported in Table 5. Classification accuracy seem mostly affected when factor SPEED = fast: 75 and 62.5% of the participants shows a worsened performance for factor COLOR = white and COLOR = green respectively, regardless of stimulus type. The number of subjects affected by change in accuracy is reduced when factor SPEED = slow, nevertheless performance can be more dramatically affected and reach 100% reduction.

**Table 5:** Influence of P_*z*_, PO_7_, and PO_8_ on classification results.

	**Speed** = **FAST**				**Speed** = **SLOW**			
**Subject**	**WH-CH**	**GR-CH**	**WH-IC**	**GR-IC**	**WH-CH**	**GR-CH**	**WH-IC**	**GR-IC**
S1	60 (40.0)	80 (20.0)	100 (0.0)	100 (0.0)	100 (0.0)	100 (0.0)	100 (0.0)	100 (0.0)
S2	20 (75.0)	100 (0.0)	60 (40.0)	100 (0.0)	100 (0.0)	100 (0.0)	100 (0.0)	100 (0.0)
S3	80 (20.0)	100 (0.0)	20 (80.0)	60 (40.0)	100 (0.0)	100 (0.0)	40 (60.0)	80 (20.0)
S4	40 (33.3)	80 (20.0)	40 (0.0)	60 (40.0)	60 (40.0)	80 (20.0)	100 (0.0)	100 (0.0)
S5	40 (60.0)	20 (80.0)	40 (33.3)	80 (20.0)	0 (100.0)	20 (66.7)	20 (66.7)	0 (100.0)
S6	100 (0.0)	80 (20.0)	60 (40.0)	60 (40.0)	100 (0.0)	100 (0.0)	100 (0.0)	100 (0.0)
S7	100 (0.0)	100 (0.0)	20 (75.0)	100 (0.0)	100 (0.0)	100 (0.0)	100 (0.0)	100 (0.0)
S8	60 (25.0)	40 (50.0)	20 (50.0)	20 (75.0)	60 (25.0)	80 (20.0)	80 (20.0)	100 (0.0)

*Data refers to the selection accuracy achieved using the reduced data set, i.e., excluding P_z_, PO_7_, and PO_8_. All values are expressed as percentages, in parentheses is reported the reduction with respect to the selection accuracy obtained using the whole electrode set (0.0 means no reduction). Stimulus type: CH, character-speller, IC, icon-speller; Stimulus color: WH, white, GR, green*.

### 3.5. Usability assessment

**Figure 7:**
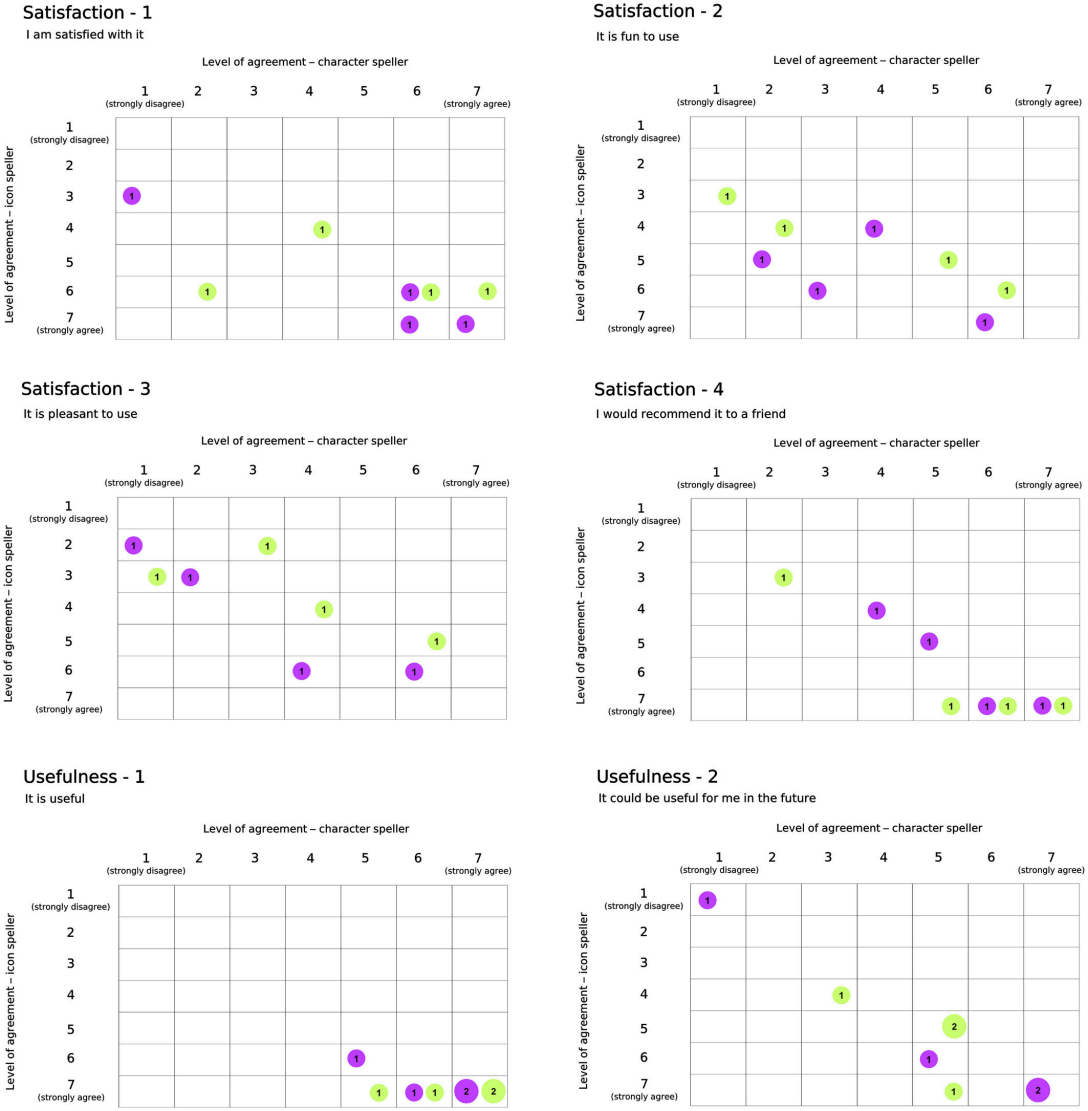
Usability Questionnaire, results related to domains Satisfaction (4 sentences) and Usefulness (2 sentences). For each sentence is reported a confusion matrix, where rows and columns refer to the level of agreement for icon and character-speller respectively. Bubbles represent joint frequencies, with diameter proportional to the number of respondents. Bubble colors refer to experimental group: violet, CH-IC and green, IC-CH.

**Figure 8:**
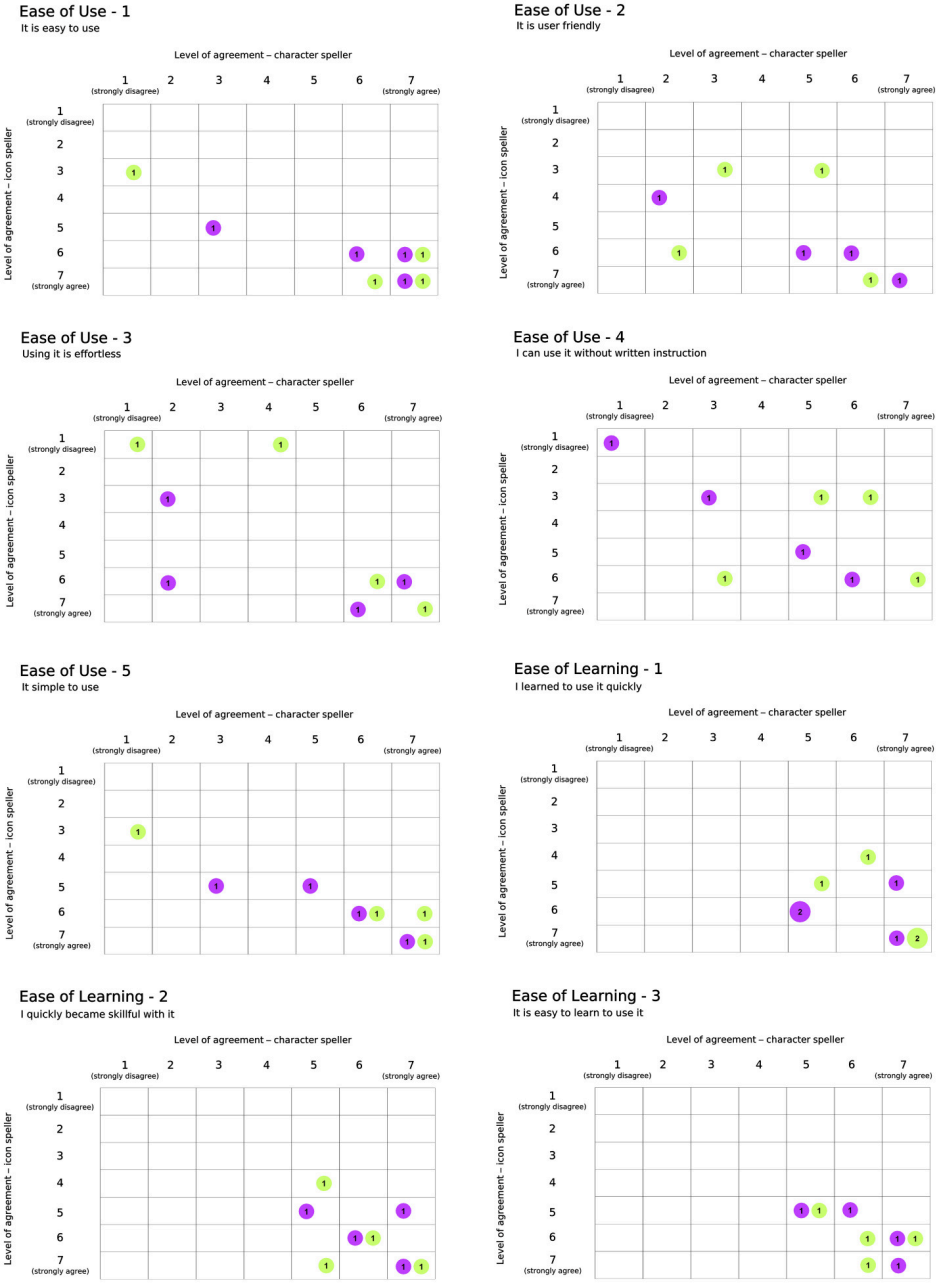
Usability Questionnaire, results related to domains Ease of Use (5 sentences) and Ease of Learning (3 sentences). Conventions are the same as in Figure 7.

Results with respect to the Usability questionnaire are reported in Figures 7, 8, which highlights changes in the level of agreement expressed by participants after their BCI experiences. Each plot depicts the confusion matrix for the two groups. Rows and columns refer to the level of agreement expressed after using the icon- and the character-speller, respectively. Thus, bubbles represent joint frequencies for each row/column combination, with a diameter proportional to the absolute joint frequency (also reported within the bubble). The color is related to the group: *violet* for the group CH-IC and *green* for the group IC-CH. Let's consider, for instance, the subplot related to the statement “It is useful” (domain: Usefulness, Figure 7), for which all participants expressed a level of agreement above “indifferent” (i.e., greater than 4): we can notice that the level of agreement was higher after using the icon-speller, regardless of the experimental sequence and that those who did not change their agreement are equally divided between the sequences. Overall, participants found neither speller particularly pleasant to use: 75 and 62.5% of participants expressed a level of agreement from “indifferent” (i.e., equal to 4) to “strongly disagree” with the statement “*It is pleasant to use*” for character- and icon-speller respectively. Nevertheless, they were quite satisfied with both spellers (62.5 and 75% of participants expressed a level of agreement above “indifferent” (i.e., greater than 4) for character- and icon-speller respectively) and found icon-speller more fun to use than character-speller (Figure 7, domain: Satisfaction). They perceived the character-speller as slightly easy to learn than the icon-speller (Figure 8, domain: Ease of learning) and rated both BCI-spellers as not particularly effortless to use (Figure 8, domain: Ease of use).

## 4. Discussion

The purpose of the present study was to analyze the within-subjects variability in P300-BCI speller performances with respect to both a character-speller and an icon-speller. We aimed at:

- evaluating whether the issues in using the icon-speller reported for a sample of disabled subjects are also observed in the present sample of able-bodied participants,
- disentangling the visual cognition process underlying end-user experience with a character- or an icon-speller by means of an electrophysiological approach.

Hence, we designed a within-subject study interlacing a cross-over approach with a factorial one. We considered two different 6×6 speller matrices: a standard character-speller (factor STIM = CH) and an icon-based one (factor STIM = IC). We also varied both stimulus COLOR (factor COLOR: white or green) and stimulus rate (factor SPEED: fast or slow). In order to allow comparisons with results on disabled subjects reported in Carabalona et al. (2012), the same combination white×fast as well as the same classifier (LDA) were chosen. Nevertheless, because of the long duration of the whole data acquisition session, we used shorter words and icon-strings to spell. The following subsections discuss the results with respect to the aims of the study.

### 4.1. Different end-users, same issues

Neuropsychological assessments in the present sample indicate no critical impairment in working memory and visual attention. The same held for participants in the sample of Carabalona et al. (2012) and none of the end-users in either sample had previous experience in BCI-speller use. This allows a comparison reasonably not biased by cognitive impairments or previous BCI expertise.

Present results about time needed to scan still (i.e., not flashing) speller matrices also parallel those reported in Carabalona et al. (2012), confirming that the scan and comprehension of symbols embedded in the icon-speller matrix was more demanding. In the present research we added the Bells Test as a validation for the selective and sustained visual attention component specifically for the icon-speller: Spearman's ρ correlation between timing for the Bells Test and time needed to scan still speller matrices, although not statistically significant, suggests a difference in perceptual and cognitive activity when scanning the character- or the icon-speller.

Results reported in Figure 3 concern on-line selection accuracy for the different spellers, and clearly show that participants in the present study are more likely to experience an unsatisfactory interaction when using the white×fast icon-speller. Moreover, results indicate that this difficulty is mitigated for COLOR = white with slow stimulation timing and for both levels in SPEED factor when using COLOR = green. Since the experimental timeline is embedded in the way results are depicted in Figure 3, it is apparent that the drop in performance occurs whatever the experimental sequence is (*CH-IC* or *IC-CH*, i.e., first character-speller and then icon-speller or the other way around), in accordance with the results reported in Carabalona et al. (2012). Unlike their results, however, the observed performance drop for the white×fast spellers seems less pronounced in the present sample and results are not statistically significant. Hence, we could argue that, for the subjects in the present sample the two speller experiences are not as dramatically diverse from each other as for participants in the sample of Carabalona et al. (2012). Nevertheless, the issue of within-subjects BCI illiteracy observed in Carabalona et al. (2012) is also present here. Thus, the studies actually share the critical point: other things being equal, in both samples some end-users can experience unsatisfactory use of one BCI-speller (mainly the icon-speller), while having a satisfactory experience with the other one (mainly the character-speller). In other words, a well performing end-user with one speller type can turn into a bad and very unsatisfied one with another speller, learned in the same way, but based on a different set of symbols.

With respect to Usability evaluation, participants rated the icon-speller as more useful and fun to use than the character-speller in accordance with the findings reported in Carabalona et al. (2012) on disabled subjects. Contrary to the opinions expressed by present end-users' group, those end-users rated the character-speller as slightly more easy and satisfying to use than the icon-speller. In explaining this difference in agreement, we should consider that participants in the study of Carabalona et al. (2012) experienced only white×fast spellers. Thus, since subject's opinion could be biased by his/her performance with BCI-spellers, ratings expressed by participants in present study could have been influenced by the better performance they achieved with the icon-spellers with green stimuli. Finally, participants rated both spellers as not particularly effortless or pleasant to use, whereas subjects in Carabalona et al. (2012) found character-speller slightly effortless and both spellers quite pleasant to use. In this case, the disagreement could be explained with the use of both fast and slow stimulation timing, which led to longer acquisition times and this is certainly more boring and demanding than the use of fast flashing BCI-spellers only.

### 4.2. Electrophysiological markers of visual cognition process underlying end-user experience with BCI-spellers

Unsatisfactory use of the white×fast icon-speller with respect to the white×fast character-speller was observed also in the present sample. Thus, there is experimental evidence suggesting stimulus type as a source of within-subjects performance variability and consequent within-subjects BCI-illiteracy. Given the visual nature of the task associated with a BCI-speller and the experimental evidence that we automatically categorize visual items (Greene and Fei-Fei, 2014), we looked for electrophysiological markers of visual object categorization during BCI spelling considering two phases: training (learning phase for end-user and classifier, thus no useful feedback is provided by the BCI system) and performance (real usage with meaningful feedback provided by the BCI system).

#### 4.2.1. Off-line ERP analysis − slow stimulation timing

In order to avoid confounding related to ERP distortion effects arising with fast stimulation timing (flash time 60 ms, dark time 10 ms), we performed off-line analysis of target-ERP data related to the different speller types under slow stimulation timing condition (flash time 100 ms, dark time 900 ms) for both training and performance phase.

With respect to the **training phase**, we observed a statistically significant difference in posterior activity (N1 component for P_*z*_ and PO_7_) highlighting more perceptual activity for icons than for characters, which seems not to be related to COLOR type. Furthermore, about 200 ms post stimulus, a divergence in C_*z*_ activity between ERPs for icons and characters emerges, although with no sufficient evidence for a STIM main effect for the P300 peaking on C_*z*_.

Results of ERP data analysis related to the **performance phase** showed a statistically significant effect for PO_8_ but not for PO_7_, indicating a right lateralization in the posterior activity with respect to the training phase. Moreover, we observed on PO_8_ a N1 component more pronounced for characters than for icons, contrary to the findings for the N1 component on PO_7_ with respect to the training phase.

In the present study, subjects carried out the performance phase always after the training phase. The critical difference between the two phases is that, during the training phase, each symbol to spell was followed by a meaningless feedback (the symbol “@,” defined as meaningless because it is not present in the speller matrix), whereas during the performance phase the participant received a meaningful feedback. This makes the nature of the perceptual interaction with each symbol (character or icon) less *morphological* and more *functional*: the selection of one symbol is now really aimed at spelling a word or at interacting with the environment. The provided feedback is indeed meaningful because, after the on-line selection made by the classifier embedded in the BCI-speller, the selected symbol is printed on the PC-screen in front of the subject spelling the word and, in case of the icon speller, resulted also in an action in the smart home. This transition from morphological to functional is a key element for real-world expertise as pointed out in James and Cree (2010). In their review about object recognition and expertise, they report about the difference between birders and ornithologist: since the birders identify the birds in the field, they are known to have a pragmatic knowledge of the birds and their parts as opposed to ornithologist, more trained in theoretical settings about morphological differences. Gathering the role of the BCI-feedback in the transition from a less morphological to a more functional perceptual interaction with the fact that participants in the present study can be considered real-world experts with respect to characters and novices to icons, it becomes clear that real-world expertise is relevant in discussing how present results about training and performance phase parallel those of Tanaka et al. (1999) and Tanaka and Curran (2001). In those studies, subjects didn't receive any special training in the Lab and, after reading a list of items, were asked to categorize items at different semantic levels without feedback. Moreover, only in the second sample, subjects are explicitly engaged in their domain of expertise.

During the training phase with a BCI-speller, subjects are trained to perform the cognitive task required by the BCI paradigm and, given the prevailing morphological nature of the visual interaction with the speller items, they are engaged in a perceptual task as in Tanaka et al. (1999). Thus, the congruence with the findings reported in Tanaka et al. (1999) and our results suggest that participants categorized characters at the basic and icons at the subordinate level. Our findings are also corroborated by the remarks of Palmeri and Gauthier (2004), who point out that letters are identified without considering font and writing-style variations, thus they are categorized at the basic and not at the subordinate level. During the performance phase, subjects are instead more explicitly engaged in their domain of expertise as in Tanaka and Curran (2001), given that the BCI-speller usage is mediated by a meaningful feedback making the perceptual interaction more functional. Our findings parallel those reported in Tanaka and Curran (2001) about real-world experts (bird- and dog-watchers). Authors report both a right lateralization and an enhancement in the N1 component when participants were asked to categorize items in their domain of expertise relative to when the task was to categorize items outside their domain of expertise. Therefore, we can argue that the more pronounced N1 component observed on PO_8_ for characters is a marker for the real-world expertise whereas the lesser activity observed for icons is indicative of the novelty of the stimulus.

Finally, a relevant feature of BCI systems is the mutual-learning of man and machine. Thus, we should also consider the role of the feedback in fostering the learning process (Tarr and Cheng, 2003). There is experimental evidence (Apitz and Bunzeck, 2012) that training can modulate N1 amplitude when objects are categorized at the basic level (as is the case for character items) and Scott et al. (2006) showed likewise that early stages of visual category processing can be influenced by feedback during the training in the Lab. Nevertheless, as pointed out in Scott et al. (2006) and Tanaka et al. (2005), simply perceptual exposure is not enough to improve visual expertise.

#### 4.2.2. Off-line ERP analysis − fast stimulation timing

The slow stimulation timing condition, in particular the 900 ms dark time window, enabled to highlight differences in the semantic categorization levels used by subjects with respect to character- and icon-speller. Nevertheless BCI systems are expected to work faster in order to be really helpful in communicating or operating the environment: in the present study, we considered also fast stimulation timing, setting flash and dark time as in Carabalona et al. (2012) for comparison purposes. The chosen flash time (60 ms) and dark time (10 ms) are also in line with the timing currently used for BCI-spellers.

In using fast stimulation timing we have to deal with distortion of target ERP morphology, affected by both overlap and refractory effects (Martens et al., 2009). Target ERPs are also affected by the fast flashing of non-target (DiRusso et al., 2003) and the presence of steady state visual evoked potentials (SSVEP) data is acknowledged in the BCI community (Sellers et al., 2006; Treder and Blankertz, 2010; Sellers, 2013). Therefore, in case of fast stimulation, we cannot rely on time-domain methods to study N1 modulation observed for slow stimulation timing because ERP waveform is dramatically distorted. Nevertheless, it seems reasonable to assume that the cognitive process of visual stimulus categorization *per-se*, as it emerged from slow stimulation timing analysis, is the same also for the fast stimulation timing. Therefore, since our aim is to understand why some well performers can become BCI-illiterate depending on speller type, we capitalized on results obtained for slow stimulation timing and analyzed only the performance phase using a time-frequency approach. As mentioned in the Methods section, flash and dark times have been designed to be 60 ms and 10 ms respectively. Nevertheless, we have to deal with the non real-time nature of the BCI-system and with monitor refresh rate (60 Hz, i.e., every 16.66 ms). This means that the actual target stimuli exposure duration is 66.64 ms and that the actual SSVEP associated with non-target stimuli, depending from dark time also, ranges from 12 to 15 Hz. Phase locking observed for non-target data on O_*z*_, where the SSVEP is maximally expected, is in accordance with this range. Moreover, we observed a modulation of phase locking also for target data confirming the effect of fast flashing non-target also on target stimuli. With respect to wrong selections, we can distinguish medium performers (i.e., those with a selection accuracy ranging from 80 to 60%) and bad performers (i.e., those with 40% selection accuracy). For medium performers we observed a paradoxical increment of phase locking in the SSVEP range. We use the term paradoxical because SSVEP is supposed to be enhanced by attention (Morgan et al., 1996). The point here is that the SSVEP induced by the flashing of non-target items should be more similar to an irrelevant stimulus when the subject is actively engaged in the BCI task. Silberstein et al. (1990) demonstrated that the SSVEP to irrilevant flickering background (13 Hz) was reduced when subjects performed an active target detection in comparison to when no target was expected, thus the observed increment along with the wrong selection is more indicative of disengagement. Nevertheless, we observed a decrease in phase locking in the SSVEP frequency range for bad performers, which seems to be inconsistent with previous results.

We speculate that disengagement plays a key role in explaining both observed increment and reduction. When disengagement occurs the subject enters a state of mind-wandering and this has diverse implications. The first one is related to pupillary dinamics: recent results show that task-disengagement is associated with both decreased baseline pupil diameter and diminished stimulus-evoked pupil dilation (Gilzenrat et al., 2010; Hopstaken et al., 2015). The second one is related to the role of alpha rhythm in both visual perception performances and mind-wandering. Experimental evidence shows that perceptual performance is related to alpha power and phase locking. Hanslmayr et al. (2005) showed that good perception performers exhibited higher P1 and N1 as well as significant higher post stimulus phase locking in the frequency range form 8 to 14 Hz than bad performers. Moreover, Hanslmayr et al. (2005) and Ergenoglu et al. (2004) observed lower alpha amplitude in the reference (baseline) period preceding task performance. These results are interpreted in terms of cortex activation as enabling perceptual performance and are also in line with the results of Cooper et al. (2003), which relate alpha synchronization/desynchronization with internal and external directed attention. Finally, there is experimental evidence supporting the theory that mind wandering can occur at different levels instead of being a dichotomous phenomenon occurring in a all-or-none fashion (Schad et al., 2012). Gathering these results, we propose that the end-user can enter different levels of mind-wandering. Since there is partial overlapping between alpha range and SSVEP frequency range, this results in a modulation of the SSVEP effect in both target and non-target. We interpret the presence of a high SSVEP-like component in both target and non-target corresponding to wrong selections as a marker of a mild-wandering state, during which the subject is not fully engaged and therefore all stimuli (i.e., both non-target and target) contribute to the SSVEP. The reduction on the SSVEP effect with respect to worse performers should then be related to a deeper mind-wandering, a state in which the participant is disconnected to the external SSVEP.

The point is now how stimulation timing can affect subjects' engagement. A direct relation would imply that boredom and disengagement are more likely for the slow stimulation timing but results on selection accuracy show a level of homogeneity in contradiction with that assumption. Moreover, selection accuracy for fast spellers also indicate that the relation between timing and engagement is not direct and that we have to consider the interplay between stimulus type and timing, i.e., the relation between semantic categorization levels and exposure time. Experimental results indicated a different effect of exposure time on categorization levels. Jolicoeur et al. (1984) used 1,000 and 75 ms as long and short exposure times in a research involving sixteen healthy subjects in a picture naming experiment. In particular, the 75 ms threshold resulted critical for subordinate, but not for basic and superordinate categorization levels. Tanaka (2001) further discusses the entry-point concept for face recognition, showing that a short exposure time (below the 75 ms threshold) impairs categorization at subordinate level only for non-face objects. With respect to stimulus exposure for BCI-spellers, 100 ms is a typical flashing time but there is also evidence regarding faster stimulation timing: in a study involving both healthy and ALS subjects, McFarland et al. (2011) used a wide range for exposure time (from 250 to 31.25 ms) and dark time (from 125 to 15.625 ms, respectively). Nevertheless, they only considered character-spellers, proving the superiority of slower stimulation rates. Münßinger et al. (2010) and Zickler et al. (2013) performed experiments with ALS as well as healthy participants using both a classical character-speller and a Brain Painting BCI system. They used a fast stimulation timing (62.5 ms for both flashing and dark time) for either speller and results showed a drop in accuracy for the Brain Painting, which was then mitigated in a second experiment where participants used a Brain Painting interface based on different icons. Finally, new emerging paradigms for BCI-spellers based on flashing faces superimposed on characters (Kaufmann et al., 2011, 2013) use also very short exposure times (31.25 ms with 125 ms dark time) and seem to improve performances when compared to plain character-spellers. Thus, if we choose fast stimulation timing, overlap and refractory effects seem to be more responsible for performance variability in using a character-speller and, in accordance with the results of Tanaka (2001), no detrimental effect seems to emerge for fast flashing faces. But if we are using icon-spellers, the critical role of the “75 ms threshold” becomes evident. In our fast stimulation timing condition, the actual exposure time is 66.64 ms: we also considered the possibility of two consecutive target-flashes, which could led to longer exposure times (133.28 ms). Our data shows that, in the worst case, the likelihood was 20% for a trial of 30 targets. Moreover, this target “repetion effect” was not related to performance. In conclusion, by using slow stimulation timing, we prevented modifications on ERP morphology and showed that icons are categorized at a subordinate level. Short exposure time is expected to affect target ERP morphology at the same extent for both icon- and character-speller, but the under-threshold (i.e., under 75 ms) stimulation timing only impairs icons perception and, at the end, can have a detrimental effect inducing subject's disengagement.

#### 4.2.3. Influence of the information embedded the N1 component on classifier selection accuracy

We also considered the influence of the N1 component on BCI performance. Since the information carried by the N1 component is related to P_*z*_, PO_7_, and PO_8_, we assessed the effect of excluding these three electrodes on classification accuracy for both slow and fast stimulation timing. The importance of P_*z*_, PO_7_, and PO_8_ is confirmed by the results, with a more marked effect for the fast than for the slow stimulation timing. Results for slow stimulation timing highlight that well performers can rely also only on the P300, but also that N1 becomes relevant for medium to bad performers. This means that EEG data in well performers lead to well separated classes, which can overcome the loss if information occurring when we use the reduced electrode set. The same is not true for medium to bad performers. In order to clarify this, we have to recall that the selection of one item in the speller is based on the weighting of rows and columns elements in the speller matrix. Thus, the change in selection accuracy arises from a change in the weights computed in the reduced electrode set with respect to those computed for the full electrode set. Since weights are calculated as the cumulative sum (over the number of row and column flashes, respectively) of posterior probabilities for target-class membership, each weight evolves as a monotonic nondecreasing function. Thus, the more one single function (i.e., weight of one row or column) is monotonically increasing with respect to the remaining five, the better we distinguish that row (or column) from the remaining five. In case of well performers, we actually observed that the weight for the target row/column is clearly separated and monotonically prevailing over the remaining five. Reducing the electrode set erodes this gap, which is however wide enough to lead also in this case to the correct selection. In case of medium to bad performers, we observed that there is no clear monotonic prevalence of one weight on the remaining five. Excluding P_*z*_, PO_7_, and PO_8_ has therefore a more dramatic effect, since it erodes an already narrower gap and can more likely lead to a wrong classification.

Finally, the observed reductions in case of fast stimulation timing seem to be more related to stimulus color than to stimulus type. Since we considered both parietal and parieto-occipital electrodes, we actually eliminated information related to both the dorsal and ventral pathways, thus reducing the mitigation effect of COLOR observed for selection accuracy obtained for the whole electrode set.

### 4.3. Study limitations

Some limitations exist in our study. Notwithstanding that participants are balanced with respect to demographic characteristics, our sample size is the minimum required by the study design. Thus, although we managed statistical analysis according to a non parametric approach and used permutational methods with correction for multiple testing, the small sample size limits the strength of our results. A different and more “intrinsic” limitation is related to the stratified and diverse analysis of electrophysiological data we performed according to SPEED factor. We decided to evaluate the two levels separately because we had to take into account the distortion effects arising in case of fast stimulation timing. Therefore, even if this choice prevented us from exploring interactions between the factor SPEED and the other two factors, we were able to control the misleading picture originating from the notable differences in epoched EEG data for the two levels of stimulation timing.

## 5. Conclusions

Despite research efforts, variability in performance and BCI-illiteracy are still critical issues for real world BCI applications. A quite unaddressed kind of BCI-illiteracy becomes apparent when the same end-users operate spellers aimed at different applications. In the present study, we considered a sample of healthy subjects using both character- and icon-spellers. We designed a within-subjects study interlacing a cross-over approach with a factorial one and manipulated stimuli type (factor STIM: characters, icons), color (factor COLOR: white, green) and timing (factor SPEED: fast, slow). For the combination fast×white, performance worsening for the icon-speller with respect to the character-speller seems to be independent from end-users' sample, since results are consistent with those reported in a previous study on disabled subjects using the same speller types. The worsening in on-line classification accuracy seems to be mitigated for fast spellers when COLOR = green or when using slow spellers. Moreover, the use of slow stimulation timing shed a new light on the perceptual and cognitive phenomena related to the use of a BCI-speller and their neurophysiological bases. Regardless of the factor COLOR, we observed visual cognition effects related to the use of either speller. Our findings are consistent with existing results on ERP tracking of object categorization and indicate that participants used different semantic categorization levels: subordinate for icons, and basic for characters. This difference in categorization levels becomes critical in case of fast stimulation timing, where an exposure time threshold (75 ms) for effective subordinate level categorization for non-face objects seems to exist. Thus, the role of this threshold becomes critical for icon-spellers: flashing stimuli categorized at the subordinate level under the “75 ms threshold” could be detrimental for end-user's performance because this categorization level is more perceptually demanding. Moreover, subjects' disengagement effects might occur because of the perceptual difficulty. Hence, for a BCI-speller to be usable, it is relevant to take into account also at which semantic level the items used in the speller matrix are categorized and the interplay between stimulus type and timing.

Our findings are also in line with growing methodological evidence about optimal electrode selection and provide some new experimental results regarding the fundamental role of parieto-occipital electrodes for BCI classifier selection accuracy, which can contribute to a deeper understanding of the “BCI-speller experience.”

## Author contributions

RC conceived and designed the study; recruited subjects and acquired the data; performed statistical analysis; interpreted results and wrote the paper.

### Conflict of interest statement

The author declares that the research was conducted in the absence of any commercial or financial relationships that could be construed as a potential conflict of interest.
